# Association Between Physician Handoffs and Major Adverse Outcomes in the Emergency Department: A Retrospective Cohort Study

**DOI:** 10.7759/cureus.117821

**Published:** 2026-10-12

**Authors:** Judicael Adadja, Akram Mokhtari, David Simonyan, Myriam Mallet, Sébastien Blais, Denis Talbot, Lynne Moore, Simon Berthelot

**Affiliations:** 1 Population Health and Optimal Health Practices Research Unit, Centre de Recherche du CHU de Québec-Université Laval, Québec, CAN; 2 Department of Social and Preventive Medicine, Faculté de Médecine, Université Laval, Québec, CAN; 3 Department of Medical Genetics, Centre Hospitalier Universitaire Sainte-Justine, Montréal, CAN; 4 Data Valorization and Exploitation Center, CHU de Québec-Université Laval, Québec, CAN; 5 Department of Family Medicine and Emergency Medicine, Faculté de Médecine, Université Laval, Québec, CAN

**Keywords:** canada, emergency physicians, emergency service hospital, hospital mortality, intensive care units, length of stay, patient handoffs, québec

## Abstract

Introduction

Handoffs between emergency physicians are common and suspected to contribute to communication errors, yet their association with clinical outcomes remains poorly documented. This study aimed to evaluate the association between physician handoffs and in-hospital mortality, intensive care unit (ICU) admission, and hospital length of stay among patients hospitalized following an emergency department (ED) visit.

Methods

A multicenter retrospective cohort study was conducted among all adult patients hospitalized following a visit to any of the five EDs of the CHU de Québec-Université Laval in Québec City, Canada, between April 2016 and March 2017. Data were extracted from the hospital administrative databases (MED-ECHO and SIURGE). Analyses were performed using generalized estimating equation (GEE) models with overlap weighting based on propensity scores, allowing adjustment for confounding and accounting for repeated visits. Subgroup analyses and multiple sensitivity analyses were conducted to assess the robustness of the findings.

Results

Among the 21,136 ED visits resulting in hospitalization, 50.6% involved at least one handoff. No significant association was observed between handoffs and in-hospital mortality (adjusted risk ratio [aRR] = 1.11; 95% CI: 0.94-1.30), ICU admission (aRR = 1.06; 95% CI: 0.91-1.23), or hospital length of stay (adjusted geometric mean ratio [aGMR] = 1.01; 95% CI: 0.96-1.08). Results were consistent across all subgroup and sensitivity analyses.

Conclusion

Handoffs between emergency physicians were not associated with an increased risk of death, ICU admission, or prolonged length of stay. Improvement efforts should focus on other components of ED operations.

## Introduction

In medical practice, continuous care requires rotating teams and inevitably leads to end-of-shift information transfers, commonly referred to as handoffs. A physician handoff refers to the process through which the clinical responsibility and accountability for a patient, or a group of patients, are transferred from an outgoing physician to an incoming one [1]. This moment represents a critical step in the care trajectory, as the quality of the transfer may influence continuity, safety, and the overall quality of patient management [2].

Handoffs are particularly frequent in the emergency department (ED), a setting characterized by high patient flow, substantial workload, wide variability in case severity, and a highly stressful environment [3]. Moreover, patient care is often fragmented among several physicians, further increasing the complexity of the process. In such a context, maintaining a clear, complete, and structured handoff is a major challenge. This complexity heightens the risk of incomplete or inadequate handoffs, potentially compromising continuity of care. According to the Joint Commission, a U.S.-based healthcare accreditation organization, failures related to handoffs are among the leading causes of serious adverse events and may be implicated in more than half of reported incidents [4]. In EDs, up to 24% of malpractice claims are attributed to errors occurring during handoffs [4], and 30% to 45% of handoffs reportedly omit essential information [5-7]. Such gaps may result in care discontinuity, reduced patient satisfaction, diagnostic and therapeutic errors, delays in treatment, clinical deterioration, potentially avoidable intensive care unit (ICU) admissions, and death [8,9]. 

Although these observations highlight the importance of the issue, evidence regarding the effect of handoffs on clinical outcomes remains limited. While other organizational factors in the ED, such as crowding or delays in care, have been clearly associated with increased mortality and complications [10-11], the specific effect of handoffs has been far less documented. This lack of data represents an important gap given the central role of information transfers in an environment where continuity of care is particularly vulnerable. To help address this gap, the present study aimed to evaluate the association between physician handoffs and in-hospital mortality, ICU admission, and hospital length of stay among patients hospitalized following an ED visit.

## Materials and methods

Study setting and design

A multicenter retrospective cohort study was carried out in the EDs of the five hospitals of the CHU de Québec-Université Laval in Québec City, Canada. The CHU de Québec-Université Laval is one of the three largest academic hospital networks in Canada. Together, these EDs manage more than 230,000 annual visits and serve a catchment population of approximately two million individuals across eastern Québec and northwestern New Brunswick [12]. The CHU de Québec-Université Laval delivers a comprehensive continuum of care, ranging from primary services to highly specialized tertiary and quaternary care. Ethical approval for the study was obtained from the Research Ethics Board of the CHU de Québec-Université Laval (approval number 2018-3848).

Participants

The study population included all adults aged 18 years or older who were hospitalized following a visit to any of the EDs of the CHU de Québec-Université Laval between April 2016 and March 2017. Eligible visits were those for which the first medical assessment in the ED was performed by an emergency physician and that subsequently resulted in hospital admission, regardless of the reason for consultation. Patients initially assessed at another healthcare facility and secondarily transferred to one of the study sites were excluded.

Study variables

Outcomes

The primary outcome was in-hospital mortality, defined as any death occurring between hospital admission and discharge. Two secondary outcomes were considered. The first was admission to the ICU during the hospitalization following the ED visit. The second was hospital length of stay, calculated in days from the time the patient left the ED for inpatient admission until discharge from the hospital.

Exposure

The exposure of interest was the occurrence of a handoff between emergency physicians. A handoff was defined as the transfer of clinical responsibility from an outgoing physician to an incoming one when the ED care episode extended across at least two consecutive work shifts. The ED care episode was defined as the interval between the patient’s first assessment by the ED physician and the first assessment by the admitting specialist. A handoff was classified as present only when the admitting specialist did not assess the patient within the first hour of the incoming shift, thereby ensuring that clinical responsibility had effectively transitioned to the incoming ED physician. We considered that a patient assessed by an admitting specialist within the first hour after handoff had not undergone a true transfer of responsibility between emergency physicians, as the patient had already been taken over by another treating team. This operational definition reflects the organizational structure of ED workflows within the participating hospitals and is consistent with the way handoffs are represented in the study data (Figure 1).

**Figure 1:**
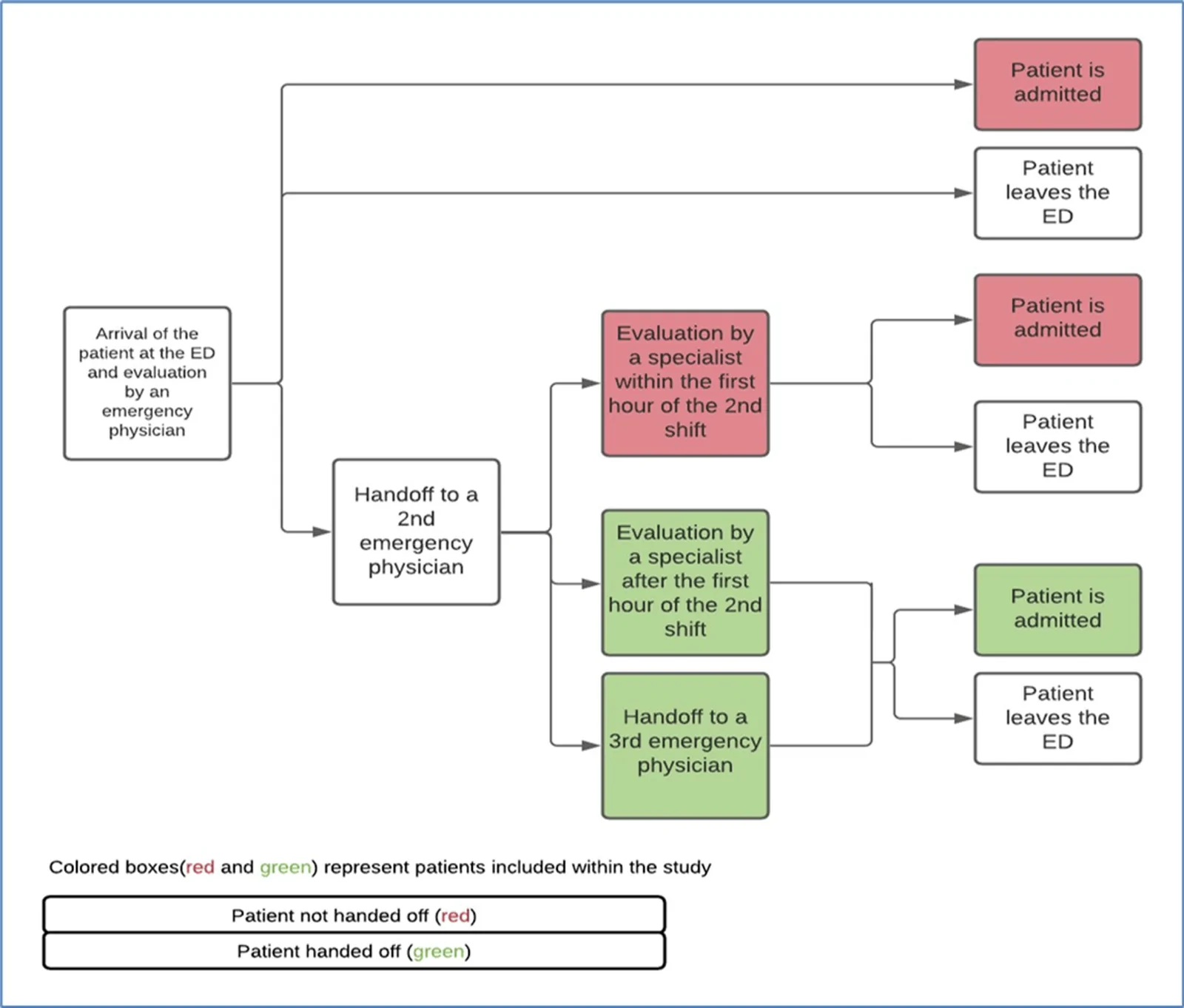
Flowchart of the possible clinical pathways for a patient within the study

Covariates

Covariates included variables that were potentially confounding as well as those constituting common causes of both hospitalization and outcomes, in order to control for confounding bias and potential collider stratification bias. Patient-level covariates included age, sex, and the Charlson Comorbidity Index. Additional covariates described ED visit characteristics, including arrival shift (day, evening, or night), Canadian Triage and Acuity Scale (CTAS) level (ranging from 1 to 5, with 5 indicating the lowest acuity), time to first medical assessment by an emergency physician, time to evaluation by a consulting specialist, initial patient orientation in the ED, ED length of stay, and hospital site. Two indicators of ED operational pressure were also included: ED occupancy ratio (patient-hours per bed) and ED arrival rate (new presentations per hour per bed).

Data Sources

Data for this study were extracted and linked by the Clinical and Organizational Performance Management Unit of the CHU de Québec-Université Laval, which routinely contributes to performance evaluation initiatives at the local, regional, and national levels. Two clinical and administrative databases, MED-ECHO and SIURGE, were used for this purpose.

The MED-ECHO database contains comprehensive information on all hospitalizations in the province of Québec. It provides sociodemographic characteristics, comorbidities, admitting services, and discharge diagnoses [13]. The SIURGE database includes detailed information related to ED operations, including CTAS level, assessment times, diagnostic and therapeutic interventions, admission requests, and ED discharge diagnoses [14].

Data from both sources were linked using the Québec health insurance number (RAMQ), a unique identifier that allows reconstruction of each patient’s complete care trajectory with a high degree of reliability. This linkage process enabled the creation of an integrated dataset containing all variables necessary to describe the ED care episode, determine exposure status, and analyze clinical outcomes.

Study size

Sampling was based on the exhaustive inclusion of all eligible visits occurring between April 2016 and March 2017. A total of 21,136 visits met the eligibility criteria and were retained for analysis.

Statistical analysis

After data extraction and linkage, the final dataset was verified, cleaned, and subjected to consistency checks. An initial descriptive analysis was conducted according to exposure status. Patient-level characteristics were summarized based on the number of unique patients, whereas visit-level characteristics were reported using the total number of ED visits. Continuous variables were presented using the median and interquartile range, while categorical variables were summarized using absolute and relative frequencies.

The association between handoff and each outcome was estimated using generalized estimating equations (GEE) with an exchangeable correlation structure and patients as the clustering unit to account for repeated visits [15]. For in-hospital mortality and ICU admission, a Poisson distribution was used to estimate relative risks [16]. Hospital length of stay was log-transformed and analyzed using a Gaussian distribution, with exponentiated coefficients interpreted as geometric mean ratios (GMRs).

Adjustment for confounding was performed using overlap weighting based on propensity scores [17,18]. For each visit, a propensity score representing the probability of being exposed to a handoff was estimated using logistic regression, with handoff status as the dependent variable and all predefined covariates described above as predictors. Overlap weights were defined as 1 - p(x) for exposed visits and p(x) for unexposed visits, where p(x) denotes the propensity score. This approach emphasizes observations with similar exposure probabilities, reduces the influence of extreme weights, and generates a pseudo-population in which exposed and unexposed groups are more comparable, thereby mimicking key attributes of a randomized trial. Covariate balance between groups was assessed before and after weighting using standardized mean differences (SMDs), with absolute values ≤ 0.10 considered indicative of adequate balance [19].

Subgroup analyses were conducted according to age (< 65 years vs. ≥ 65 years) and triage acuity (CTAS < 3 vs. ≥ 3). Potential effect modification was assessed by testing interaction terms between handoff and each subgroup variable. The robustness of the findings was assessed through multiple sensitivity analyses. One sensitivity analysis relied exclusively on theoretical shift-change cut-off times (08:00, 16:00, and 24:00), classifying an ED care episode as involving a handoff whenever it overlapped at least two official shifts. Additional sensitivity analyses restricted in-hospital mortality and ICU admission to events occurring within 72 hours and within seven days of admission. Statistical significance was defined as p < 0.05. All analyses were performed using R version 4.5.0 (R Foundation for Statistical Computing, Vienna, Austria) [20].

## Results

Characteristics of included visits

A total of 21,136 ED visits resulting in hospitalization were included in the study. These visits were made by 17,148 unique patients during the observation period. The median patient age was 71 years (IQR: 55.0-83.0), and 6,562 (38.2%) patients were younger than 65 years. Slightly more than half were women (n=9,210; 53.7%), and 6,837 (39.9%) had a Charlson Comorbidity Index below 3.

The temporal distribution of visits showed that 10,609 (50.2%) occurred between 08:00 and 15:59, whereas 3,286 (15.5%) occurred during the nighttime period (00:00-07:59). At triage, 5,714 (27.1%) visits were classified as CTAS levels 1 or 2. The median ED length of stay was 20.8 hours (IQR, 9.8-32.6). Indicators of ED operational pressure showed a median of 0.2 new ED visits per hour per bed (IQR, 0.1-0.2) and a median ED occupancy level of 1.6 patient-hours per bed (IQR, 1.3-1.8).

Regarding exposure status, 10,693 (50.6%) visits involved at least one physician handoff. Among these, 7,328 (68.5%) involved one to two handoffs, whereas 3,365 (31.5%) involved three or more handoffs. Nearly half of all handoffs (n=5,328; 49.8%) occurred around the 08:00 shift change, while 2,151 (20.1%) occurred around the 16:00 shift change (Table 1).

**Table 1:** Characteristics of emergency department visits stratified by handoff status (17,148 unique patients; 21,136 visits) ED: emergency department; ICU: intensive care unit † Median (IQR) * Analysis based on unique patients (n = 17,148); all other variables analyzed at the visit level (n = 21,136)

Characteristics	No handoff n (%)	Handoff n (%)	Overall n (%)
Overall	10,443 (49.4)	10,693 (50.6)	21,136 (100.0)
Age (years)^*†^	68.0 (52.0, 81.0)	73.0 (58.0, 84.0)	71.0 (55.0, 83.0)
Age group^*^			
18-49	1,940 (22.7)	1,375 (16.0)	3,315 (19.3)
50-64	1,771 (20.7)	1,476 (17.2)	3,247 (18.9)
65-74	1,709 (20.0)	1,663 (19.4)	3,372 (19.7)
75-85	1,672 (19.5)	2,056 (23.9)	3,728 (21.7)
85+	1,468 (17.1)	2,018 (23.5)	3,486 (20.3)
Sex^*^			
Female	4,317 (50.4)	4,893 (57.0)	9,210 (53.7)
Male	4,243 (49.6)	3,695 (43.0)	7,938 (46.3)
Charlson Comorbidity Index^*^			
0	1,749 (20.4)	1,220 (14.2)	2,969 (17.3)
1-2	2,114 (24.7)	1,754 (20.4)	3,868 (22.6)
3-4	3,245 (37.9)	3,615 (42.1)	6,860 (40.0)
5+	1,452 (17.0)	1,999 (23.3)	3,451 (20.1)
Arrival shift			
00:00-07:59	1,536 (14.7)	1,750 (16.4)	3,286 (15.5)
08:00-15:59	6,496 (62.2)	4,113 (38.5)	10,609 (50.2)
16:00-23:59	2,411 (23.1)	4,830 (45.2)	7,241 (34.3)
Canadian Triage and Acuity Scale (CTAS)			
Level 1	1,054 (10.1)	335 (3.1)	1,389 (6.6)
Level 2	2,392 (22.9)	1,933 (18.1)	4,325 (20.5)
Level 3	4,417 (42.3)	5,215 (48.8)	9,632 (45.6)
Level 4	2,185 (20.9)	2,991 (28.0)	5,176 (24.5)
Level 5	395 (3.8)	219 (2.0)	614 (2.9)
ED arrival rate (new presentations per hour per bed)^†^	0.2 (0.2, 0.3)	0.2 (0.1, 0.2)	0.2 (0.1, 0.2)
ED occupancy ratio (patient-hours per bed)^†^	1.6 (1.4, 1.8)	1.5 (1.3, 1.7)	1.6 (1.3, 1.8)
Delay to ED physician assessment (hours)^†^	0.7 (0.4, 1.3)	1.0 (0.6, 1.7)	0.8 (0.5, 1.5)
Delay between ED physician and specialist assessment (hours)^†^	2.4 (1.3, 3.9)	12.7 (8.1, 17.4)	5.2 (2.4, 12.8)
ED length of stay (hours)^†^	11.3 (6.5, 26.4)	25.5 (17.8, 42.4)	20.8 (9.8, 32.6)
Initial orientation in the ED			
Ambulatory area	56 (0.5)	8 (0.1)	64 (0.3)
Standard stretcher bay	7,991 (76.5)	9,739 (91.1)	17,730 (83.9)
Resuscitation bay	2,396 (22.9)	946 (8.8)	3,342 (15.8)
Admitting specialty			
Surgery	3,099 (29.7)	2,505 (23.4)	5,604 (26.5)
Medicine	7,026 (67.3)	6,997 (65.4)	14,023 (66.3)
Family medicine	318 (3.0)	1,191 (11.1)	1,509 (7.1)
Hospital length of stay (days)^†^	6.0 (3.0, 13.0)	6.0 (3.0, 15.0)	6.0 (3.0, 14.0)
Admission to ICU	1,407 (13.5)	642 (6.0)	2,049 (9.7)
In-hospital mortality	981 (9.4)	837 (7.8)	1,818 (8.6)

Standardized mean differences before overlap weighting are presented in Table 2. After overlap weighting, all measured baseline covariates achieved excellent balance, with absolute standardized mean differences <0.001 for all variables.

**Table 2:** SMD of baseline characteristics before overlap weighting After overlap weighting, absolute standardized mean differences were <0.001 for all measured baseline covariates. SMD: standardized mean difference; CTAS: Canadian Triage and Acuity Scale; ED: emergency department; CHUL: Centre hospitalier de l’Université Laval; HDQ: Hôtel-Dieu de Québec; HEJ: Hôpital de l’Enfant-Jésus; HSFA: Hôpital Saint-François d’Assise; HSS: Hôpital du Saint-Sacrement

Variable	SMD before weighting
Age	0.228
Sex (Male)	-0.064
Charlson Comorbidity Index	0.214
Arrival shift	
00:00-07:59	0.017
08:00-15:59	-0.237
16:00-23:59	0.221
Canadian Triage and Acuity Scale (CTAS)	
Level 1	-0.070
Level 2	-0.048
Level 3	0.065
Level 4	0.070
Level 5	-0.017
ED arrival rate (new presentations per hour per bed)	-0.514
ED occupancy ratio (patient-hours per bed)	-0.275
Delay to ED physician assessment (hours) (restricted cubic spline)	
Spline term 1	0.291
Spline term 2	0.189
Spline term 3	0.172
Initial orientation in the ED	
Ambulatory area	-0.005
Standard stretcher bay	0.146
Resuscitation bay	-0.141
Hospital	
CHUL	-0.092
HDQ	-0.002
HEJ	-0.096
HSFA	0.089
HSS	0.101
ED length of stay (hours)	0.658
Delay between ED physician and specialist assessment (hours)	1.737

Association between physician handoff and in-hospital mortality

Among the 21,136 included visits, 1,818 (8.6%) in-hospital deaths were recorded, including 837 among visits that involved at least one handoff (Table 1). Compared with visits without a handoff, those with a handoff had an 11% higher risk of in-hospital death, although this difference was not statistically significant (aRR = 1.11; 95% CI: 0.94-1.30) (Table 3). Subgroup analyses showed no significant association between handoff and mortality in either patients younger than 65 years (aRR = 1.04; 95% CI: 0.67-1.62) or those aged 65 years or older (aRR = 1.12; 95% CI: 0.95-1.33). No significant association was observed within either CTAS subgroup, with an aRR of 1.08 (95% CI: 0.83-1.40) for CTAS < 3 and 1.12 (95% CI: 0.92-1.38) for CTAS ≥ 3 (Table 3). There was no evidence of interaction by age (P for interaction = 0.751) or CTAS (P for interaction = 0.812).

**Table 3:** Association between handoff and in-hospital mortality (aRRs, N = 21,136) aRR: adjusted risk ratio; CI: confidence interval; ICU: intensive care unit; CTAS: Canadian Triage and Acuity Scale; ED: emergency department Model adjusted using overlap weights estimated from the following covariates: age, sex, Charlson comorbidity index, arrival shift, CTAS level, ED occupancy ratio, ED arrival rate, delay to ED physician assessment modeled using restricted cubic splines, initial ED orientation, hospital site, ED length of stay, and delay to specialist evaluation. Subgroup sizes were: <65 years (n = 7,634), ≥65 years (n = 13,502), CTAS <3 (n = 5,714), and CTAS ≥3 (n = 15,422).

Group	aRR	95% CI	p-value
Overall			
No handoff	1.00		
Handoff	1.11	0.94-1.30	0.214
Stratified by age			
< 65 years			
No handoff	1.00		
Handoff	1.04	0.67-1.62	0.860
≥ 65 years			
No handoff	1.00		
Handoff	1.12	0.95-1.33	0.183
Stratified by triage acuity (CTAS)			
CTAS < 3			
No handoff	1.00		
Handoff	1.08	0.83-1.40	0.583
CTAS ≥ 3			
No handoff	1.00		
Handoff	1.12	0.92-1.38	0.266

Association between physician handoff and ICU admission

A total of 2,049 ICU admissions (9.7%) were identified, including 642 among visits involving at least one handoff (Table 1). Compared with unexposed visits, those with a handoff showed a 6% higher risk of ICU admission, although the difference was not statistically significant (aRR = 1.06; 95% CI: 0.91-1.23) (Table 4). Stratified analyses by age revealed no significant association: aRR = 1.17 (95% CI: 0.95-1.45) among patients younger than 65 years, and aRR = 0.95 (95% CI: 0.77-1.18) among those aged 65 years or older. Results were similar across triage categories, with an aRR of 1.18 (95% CI: 0.98-1.42) for CTAS < 3, and 0.92 (95% CI: 0.73-1.16) for CTAS ≥ 3 (Table 4). There was no evidence of interaction by age (P for interaction = 0.168) or CTAS (P for interaction = 0.101).

**Table 4:** Association between handoff and ICU admission (aRRs, N = 21,136) aRR: adjusted risk ratio; CI: confidence interval; ICU: intensive care unit; CTAS: Canadian Triage and Acuity Scale; ED: emergency department Model adjusted using overlap weights estimated from the following covariates: age, sex, Charlson comorbidity index, arrival shift, CTAS level, ED occupancy ratio, ED arrival rate, delay to ED physician assessment modeled using restricted cubic splines, initial ED orientation, hospital site, ED length of stay, and delay to specialist evaluation. Subgroup sizes were: <65 years (n = 7,634), ≥65 years (n = 13,502), CTAS <3 (n = 5,714), and CTAS ≥3 (n = 15,422).

Group	aRR	95% CI	p-value
Overall			
No handoff	1.00		
Handoff	1.06	0.91-1.23	0.458
Stratified by age			
< 65 years			
No handoff	1.00		
Handoff	1.17	0.95-1.45	0.131
≥ 65 years			
No handoff	1.00		
Handoff	0.95	0.77-1.18	0.660
Stratified by triage acuity (CTAS)			
CTAS < 3			
No handoff	1.00		
Handoff	1.18	0.98-1.42	0.077
CTAS ≥ 3			
No handoff	1.00		
Handoff	0.92	0.73-1.16	0.494

Association between physician handoff and hospital length of stay

The median hospital length of stay was six days in both groups (Table 1). On average, visits involving at least one handoff had a hospital stay that was 1% longer than visits without a handoff, a difference that was not statistically significant (aGMR = 1.01; 95% CI: 0.96-1.08) (Table 5). Subgroup analyses showed no significant association between handoff and hospital length of stay in either patients younger than 65 years (aGMR = 1.02; 95% CI: 0.93-1.12) or those aged 65 years or older (aGMR = 1.01; 95% CI: 0.94-1.09). No significant association was observed within either CTAS subgroup, with an aGMR of 1.06 (95% CI: 0.95-1.19) for CTAS < 3 and 1.00 (95% CI: 0.93-1.07) for CTAS ≥ 3 (Table 5). There was no evidence of interaction by age (P for interaction = 0.877) or CTAS (P for interaction = 0.322).

**Table 5:** Association between handoff and hospital length of stay (aGMRs, N = 21,136) aGMR: adjusted geometric mean ratio; CI: confidence interval; ICU: intensive care unit; CTAS: Canadian Triage and Acuity Scale; ED: emergency department Model adjusted using overlap weights estimated from the following covariates: age, sex, Charlson comorbidity index, arrival shift, CTAS level, ED occupancy ratio, ED arrival rate, delay to ED physician assessment modeled using restricted cubic splines, initial ED orientation, hospital site, ED length of stay, and delay to specialist evaluation. Subgroup sizes were: <65 years (n = 7,634), ≥65 years (n = 13,502), CTAS <3 (n = 5,714), and CTAS ≥3 (n = 15,422).

Group	aGMR	95% CI	p-value
Overall			
No handoff	1.00		
Handoff	1.01	0.96-1.08	0.634
Stratified by age			
< 65 years			
No handoff	1.00		
Handoff	1.02	0.93-1.12	0.644
≥ 65 years			
No handoff	1.00		
Handoff	1.01	0.94-1.09	0.735
Stratified by triage acuity (CTAS)			
CTAS < 3			
No handoff	1.00		
Handoff	1.06	0.95-1.19	0.283
CTAS ≥ 3			
No handoff	1.00		
Handoff	1.00	0.93-1.07	0.906

Sensitivity analyses

Sensitivity analyses yielded results consistent with the primary analyses. When physician handoff was alternatively defined using theoretical shift-change times, no statistically significant associations were observed with in-hospital mortality (aRR = 1.05; 95% CI: 0.92-1.20), ICU admission (aRR = 0.98; 95% CI: 0.87-1.10), or hospital length of stay (aGMR = 1.03; 95% CI: 0.98-1.09) (Table 6). Similarly, restricting outcomes to events occurring within 72 hours or seven days of admission did not materially alter the findings. Physician handoff was not significantly associated with mortality within 72 hours (aRR = 1.21; 95% CI: 0.83-1.76) or seven days (aRR = 0.97; 95% CI: 0.75-1.26), nor with ICU admission within 72 hours (aRR = 1.10; 95% CI: 0.95-1.29) or seven days (aRR = 1.10; 95% CI: 0.94-1.28) (Table 7).

**Table 6:** Association between handoff, defined using shift-change cut-off times, and in-hospital mortality, ICU admission, and hospital length of stay (N = 21,136) aRR: adjusted risk ratio; aGMR: adjusted geometric mean ratio; CI: confidence interval; ICU: intensive care unit; CTAS: Canadian Triage and Acuity Scale; ED: emergency department Model adjusted using overlap weights estimated from the following covariates: age, sex, Charlson comorbidity index, arrival shift, CTAS level, ED occupancy ratio, ED arrival rate, delay to ED physician assessment modeled using restricted cubic splines, initial ED orientation, hospital site, ED length of stay, and delay to specialist evaluation.

Variable	In-hospital mortality			ICU admission			Hospital length of stay		
	aRR	95% CI	p-value	aRR	95% CI	p-value	aGMR	95% CI	p-value
No handoff	1.00			1.00			1.00		
Handoff	1.05	0.92-1.20	0.471	0.98	0.87-1.10	0.683	1.03	0.98-1.09	0.222

**Table 7:** Association between handoff and early (≤72 h) and 7-day in-hospital mortality and ICU admission (N = 21,136) aRR: adjusted risk ratio; CI: confidence interval; ICU: intensive care unit; CTAS: Canadian Triage and Acuity Scale; ED: emergency department Model adjusted using overlap weights estimated from the following covariates: age, sex, Charlson comorbidity index, arrival shift, CTAS level, ED occupancy ratio, ED arrival rate, delay to ED physician assessment modeled using restricted cubic splines, initial ED orientation, hospital site, ED length of stay, and delay to specialist evaluation.

Outcome window	In-hospital mortality			ICU admission		
	aRR	95% CI	p-value	aRR	95% CI	p-value
≤72 hours						
No handoff	1.00			1.00		
Handoff	1.21	0.83-1.76	0.327	1.10	0.95-1.29	0.211
≤7 days						
No handoff	1.00			1.00		
Handoff	0.97	0.75-1.26	0.841	1.10	0.94-1.28	0.233

## Discussion

This study, which examined more than 21,000 hospitalized ED visits across five hospitals within a major Canadian academic health network, evaluated the association of emergency physician handoffs with three clinically significant outcomes: in-hospital mortality, ICU admission, and hospital length of stay. Overall, no statistically significant association was observed for any of the outcomes assessed. These findings remained consistent across all complementary analyses, including subgroup analyses stratified by age and triage acuity, as well as sensitivity analyses based on alternative exposure definitions and narrower temporal windows. Taken together, the results suggest that, within the organizational context evaluated, handoffs do not appear to significantly influence these outcomes at the population level.

These observations contrast with the perception that handoffs inherently threaten continuity of care and increase the risk of adverse events [1,21]. Several investigations have documented the frequency of communication failures and their purported role in clinical incidents, including the work of Kachalia et al. and Kitch et al., which highlighted perceived links between suboptimal handoffs and patient harm [22,23]. However, such studies were not designed to isolate the causal effect of handoffs themselves. Conversely, some analyses more explicitly oriented toward causal inference, such as the study by Denson et al. in internal medicine, reported increased mortality associated with resident rotations [24]. Yet these findings were derived from clinical environments markedly different from the ED, where patient profiles, care processes, and the temporal dynamics of handoffs are not comparable. To our knowledge, no prior study had specifically examined this question within emergency medicine, a setting defined by rapid decision-making, fluctuating workload, and a high frequency of clinical transitions.

The absence of a statistically significant association in this study should not be interpreted as evidence that handoffs carry no risk whatsoever. Point estimates suggested modest effects, 11% for mortality and 6% for ICU admission, that did not reach statistical significance but remain clinically important. It is therefore possible that some suboptimal handoffs contribute to isolated safety incidents without producing a detectable population-level effect on the outcomes measured in this study. These findings do not diminish the importance of improving and standardizing handoff practices, or of strengthening clinical surveillance mechanisms, structured documentation processes, and the use of formalized communication tools aimed at reducing errors, delays in care, and redundant testing, as emphasized in previous work by Milano et al. [25] and Appelbaum et al. [26].

Several strengths reinforce the validity of this study. Its multicenter design enhances generalizability to real-world ED practice, while the inclusion of more than 21,000 ED visits provided substantial statistical power. The combined use of GEE models to account for repeated visits and overlap weighting to balance confounders strengthened comparability between exposed and unexposed groups. Multiple sensitivity analyses supported the robustness of the findings.

Nonetheless, certain limitations must be considered. Handoffs were not documented, and their identification relied on overlapping work shifts, introducing potential misclassification. This operational definition, although imperfect, aligns with local practice and likely reflects workflows in many North American EDs. The study did not account for the quality, structure, or content of the handoffs, elements known to vary substantially across physicians, teams, workload conditions, and case complexity. Handoffs are not a homogeneous process, and their effectiveness may be influenced by subtler organizational factors, such as team dynamics at the time of transition or the patient’s position in the sequence of cases transferred [27]. The reliance on administrative data means that findings depend on coding accuracy and data completeness. Errors in recording or classification could theoretically produce information bias that either attenuates or exaggerates associations. However, prior evaluations have shown excellent reliability of local administrative databases, reducing the likelihood of major systematic bias [28,29]. Additionally, despite adjustment for a comprehensive set of confounders, residual confounding remains possible owing to unmeasured clinical or organizational determinants not captured in administrative sources. Restricting the study population to hospitalized individuals may also have introduced collider stratification bias. However, adjustment for measured variables representing common causes of both hospitalization and outcomes may have mitigated this risk.

Finally, the results pertain exclusively to hospitalized ED patients and may not generalize to non-hospitalized ED visits, whose risk profiles and care trajectories differ substantially. External generalizability may also be limited in settings where available resources, handoff protocols, or organizational structures differ meaningfully from those examined in this study.

## Conclusions

In this multicenter study, no statistically significant adverse association was observed between emergency physician handoffs and in-hospital mortality, ICU admission, or hospital length of stay among patients hospitalized following an ED visit. The results challenge some widely held perceptions regarding the risks attributed to handoffs. Nevertheless, improving and standardizing handoff practices remains important for patient safety. Future research should aim to disentangle the respective effects of organizational and system-level factors on patient safety.
